# A stepped approach for the management of symptomatic internal derangement of the temporomandibular joint

**DOI:** 10.1186/s40463-018-0282-y

**Published:** 2018-05-15

**Authors:** Candan Efeoglu, Aylin Sipahi Calis, Huseyin Koca, Esra Yuksel

**Affiliations:** 1 Oral Surgery Department, Ege University School of Dentistry, 35100 Izmir, Turkey; 2 Anesthesiology Department, Ege University School of Medicine, 35100 Izmir, Turkey

## Abstract

**Background:**

Internal derangement is the clinical and pathological condition of disc displacement of the temporomandibular joint. Management of these cases involve conservative and surgical treatment options. Minimally invasive surgical procedures namely arthrocentesis and arthroscopy are promising techniques in the management of internal derangement. However patient selection algorithms, indications for minimally invasive procedures and details of the techniques should be further studied for safe and cost effective management of these cases.

This manuscript aims to retrospectively analyze the significance of a stepped surgical treatment approach (arthrocentesis under local anaesthesia as the first line of treatment, followed by arthroscopic lysis and lavage under general anaesthesia in unresolving cases) of internal derangement with or without osteoarthritis.

**Methods:**

This is a retrospective cohort study. Case notes of 1414 patients that were managed with a standard protocol were reviewed. Appropriate inclusion and exclusion criteria were set. Thirty-three patients were eligible for inclusion. Parameters recorded were pain-free inter-incisal opening, spontaneous pain, pain on function, difficulty on chewing, and perceived disability on jaw movements. Pre-operative and post-operative (at the end of the follow up period) pain free maximum interincisal opening values were compared with paired t test and the subjective parameters were evaluated with Chisquare analysis. Treatment outcome and success rate according to American Association of Oral and Maxillofacial Surgeons were descriptively shown.

**Results:**

Interincisal opening values increased, and the number of patients with severe or medium rated subjective parameters were reduced at discharge. These improvements were found to be statistically significant. Clinical (Wilkes) staging of internal derangement pre-operatively and at discharge remained either unchanged or was lower. Treatment outcome and success according to American Association of Oral and Maxillofacial Surgeons criteria was 94%.

**Conclusion:**

The stepped approach for the management of symptomatic internal derangement with or without osteoarthritis is a successful treatment strategy with favourable therapeutic outcomes.

## Background

Internal derangement (ID) is the clinical and pathological condition of disc displacement of the temporomandibular joint (TMJ), and the usual coexisting osteoarthritis (OA). OA is also known as osteoarthrosis or degenerative joint disease (DJD) [1].

Although a small fraction of patients with ID experience symptoms that require treatment, it is considered to be the most common cause of serious TMJ pain and dysfunction. These symptoms lead to deterioration of patients’ life quality that can necessitate surgical treatment modalities.

The prevalence of ID/OA in males and females is not well documented, but most of the patients seeking treatment are females [2–4]. Joint overloads, facial trauma, whiplash, clenching and bruxism may cause or aggravate TMJ pain and dysfunction [5, 6].

Traditional surgical treatments of ID include repositioning of the disc or discectomy. However, the introduction of minimally invasive TMJ arthroscopy in 1975 was a turning point in the surgical management of ID. [7–9]. Remarkably successful results were achieved simply with arthroscopic lysis and lavage that allowed the removal of inflammatory cytokines and lysis of adhesions limiting joint mobility [9–12].

The success of arthroscopic TMJ lavage was followed by the introduction of arthrocentesis. This technique is also shown to be effective in the treatment of ID, particularly in cases of symptomatic closed lock [10–13].

This manuscript aims to retrospectively analyze the value of our stepped surgical treatment approach (arthrocentesis as the first line of treatment, followed by arthroscopic lysis and lavage in unresolving cases) in cases that are diagnosed to have symptomatic unilateral ID with or without OA of their TMJs. The null hypothesis tested is “the stepped approach is a successful treatment strategy in the management of symptomatic internal derangements of TMJ.”

## Methods

### Study design and patient population

Case notes of patients referring to the Department of Oral Surgery in the School of Dentistry, Ege University for TMJ pain and dysfunction that were managed by the authors of this manuscript over a period of 3 years (2009–2012) were retrospectively reviewed. All the patients were managed with a standard protocol (Table 1). The local ethics committee approved this retrospective study and the treatments were carried out after obtaining verbal and written informed consents from the patients.

**Table 1:** Flow chart describing the standard treatment protocol of patients who have TMJ pain and dysfunction

1. A detailed history, evaluation of head and neck, panoramic films to demonstrate temporal bone and condylar morphology	
⇓⥥	
2. If indicated, consultations with ear, nose and throat surgery; neurology; physical medicine and rehabilitation; internal medicine; and restorative dentistry as appropriate	
⇓⥥	
3. Advices included soft diet, limiting their mouth opening and not to chew gum for 6 weeks	
⇓⥥	
4. Ibuprofen (200 mg tds for 6 weeks) was the drug of choice for TMJ pain, and if necessary muscle relaxants were added (maximum for a period of 10 days)	
⇓⥥	
5. Patients with a clenching habit were provided with mouth guards for use while sleeping	
⇓⥥	
6. Review appointment was within 6 to 8 weeks to monitor the patients’ progress.	
⇓⥥	
7. MRIs were requested from symptomatic patients with a clinical diagnosis of internal derangement. Patients with ongoing symptoms refractory to conservative treatment and MRI confirmed internal derangement were listed for arthrocentesis of the effected joint under local anaesthesia	
⥥⇓	
8. Post-operatively patients were regularly reviewed and those with persisting symptoms beyond 6 months were listed for arthroscopic lysis and lavage	
⇓⥥	
9. Post-operative reviews	

Inclusion criteria for the review process were unilateral ID with or without OA requiring surgical treatment and management according to the above given protocol. Patients who became asymptomatic by conservative measures and therefore did not require surgical intervention; patients with a history of orthognathic and/or TMJ operations, arthritis effecting other joints, bilateral TMJ problems, incomplete documentation of treatment details or inability to attend review appointments regularly, were excluded from this retrospective cohort study.

### Diagnosis and data collection

All of the patients had a panoramic film at presentation and a magnetic resonance imaging (MRI) was requested from those who had ongoing symptoms refractory to conservative treatment (Table 1). MRI was taken to confirm internal derangement. According to our standard management protocol, an objective parameter and 4 different subjective parameters were recorded pre-operatively and on post-operative reviews. Pain-free inter-incisal opening (the difference between incisal edges of central incisors when the patient is asked to open their mouth as wide as possible without any discomfort or pain) is an objective parameter and it was recorded as an average of three consecutive measurements done by the same clinician. Subjective parameters (spontaneous pain, pain on function, difficulty on chewing, perceived disability on jaw movements) were recorded pre-operatively and on post-operative reviews. For this purpose Modified VAS (visual anolgue scale) scales were printed on a scale of 4 on 4 separate sheets of paper with the subjective parameter in question as the running title and patients were asked to rate their discomfort or pain on these scales (none, mild, medium and severe). Staging (Wilkes) of internal derangement of TMJs were done according to clinical and MRI data pre-operatively and according to clinical data post-operatively [2–4]. AAOMS (American Association of Oral and Maxillofacial Surgeons) success criteria were used for post-op evaluation of outcomes and success rate.

### Treatment

Technique of arthrocentesis: Auriculotemporal nerve block is achieved and 1.5 ml of Lidocaine HCL (20 mg/ml) is used for distension of the superior joint space. A technique described by Laskin [7] was utilized for access and irrigation (with saline) of the superior joint space. 150–500 ml of saline is used for lysis and lavage. During the procedure the patient is instructed by the surgeon to carry out exercises like protrusion of the lower jaw and stretch opening the mouth. At the end of the procedure 1 ml of Orthovisc® (Biomex GmbH, Germany) is injected intra-articularly. A non-steroidal anti-inflammatory drug is prescribed post-operatively. Mouth stretching exercise to maintain the achieved range of motion is instructed [7].

Technique of arthroscopic lysis and lavage: General anaesthesia and complete neuro-muscular relaxation is achieved. After surgical preparation a 21 G cannula is used for access and distension of the joint. Later two trocar sheaths are placed from superior posterolateral and superior anterolateral approaches. Under constant saline irrigation, a 30° forward oblique telescope (Hopkins® Karl Storz GmbH & Co Germany) and a blunt obturator is used interchangibly through both access holes for sweeping the upper joint space under telescopic vision to aid lysis of the adhesions [14]. A minimum amount of 500 ml saline is used for lysis and lavage. During the procedure the jaw is manipulated to aid in the lysis of adhesions. At the end of the procedure1ml of Orthovisc® is injected intra articularly. Prescription and exercises are identical to those preferred after arthrocentesis.

### Statistical analysis

Pre-operative and post-operative (at the end of the follow up period) pain free maximum interincisal opening values were compared with paired t test. The subjective parameters and Clinical (Wilkes) staging of internal derangement pre-operatively and at discharge were evaluated with Chi-square analysis using SPSS 10.0. (SPSS Inc. Chicago, İllinois, USA) AAOMS success criteria (“parameters of care” 2017) are considered and surgical treatment outcome is calculated [15].

## Results

The authors of this manuscript managed a total of 1414 patients with TMJ pain and dysfunction between January 2009 and January 2012 according to the standard protocol outlined in Table 1. A comprehensive review of the case notes revealed that only 43 patients were surgically treated over a period of 3 years. (Arthrocentesis +/− arthroscopic lysis and lavage) According to our inclusion and exclusion criteria, 33 patients (31 females, 2 males) were regarded to be eligible for inclusion and statistical analysis. Mean age was 33.8 (range 15–63). Symptoms of 24 patients were completely or partially resolved after arthrocentesis hence did not require further treatment, however 9 patients required further treatment with arthroscopic lysis and lavage.

The follow–up period is defined as the period starting from the final surgical intervention (arthrocentesis or arthroscopic lysis and lavage) to the date of discharge. Follow-up period ranged from 3 to 30 months (mean 15.5 and standard deviation 5.8).

Pain free maximum interincisal opening values measured pre-operatively and at the end of the follow-up period were found to be statistically significant (Paired t test; *p* < 0.001) (Table 2).

**Table 2:** Mean values, standard deviation and standard error of mean for pain free maximum interincisal opening

	Mean	Standard deviation	Standard error of mean
Pre-op value (mm)	27.636	8.652	1.151
Value at discharge (mm)	33.363	6.827	1.189
Difference	5.727	7.989	1.380

Subjective parameters; spontaneous pain, pain on function, difficulty on chewing, perceived disability of jaw movements values rated pre-operatively and at discharge were found to be statistically significant (Chi-square analysis; *p* < 0.001) (Table 3 and Table 4).

**Table 3:** Distribution of patients’ subjective parameter ratings pre-operatively. (Total *N* = 33)

Subjective parameters	None (n)	Mild (n)	Medium (n)	Severe (n)
Spontaneous pain	11	3	10	9
Pain on function	0	3	15	15
Difficulty on chewing	1	5	12	15
Perceived disability of jaw movements	1	6	12	14

**Table 4:** Distribution of patients’ subjective parameter ratings at discharge (Total *N* = 33)

Subjective parameters	None (n)	Mild (n)	Medium (n)	Severe (n)
Spontaneous pain	28	3	1	1
Pain on function	27	5	1	0
Difficulty on chewing	26	5	2	0
Perceived disability of jaw movements	22	4	7	0

The number of patients with severe or medium rated subjective parameters pre-operatively was reduced at discharge. This reduction was from 57.6 to 6.1% for spontaneous pain; from 90.9 to 3% for pain on function; from 81.8 to 6.1% for difficulty on chewing; and from 78.8 to 21.2% for perceived disability of jaw movements.

Clinical (Wilkes) staging of internal derangement pre-operatively and at discharge is shown in Table 5. Accordingly clinical staging of;

**Table 5:** Distribution of patients’ clinical staging (Wilkes) of internal derangement pre-operatively and at discharge (Total *N* = 33)

	Stage I	Stage II	Stage III	Stage IV	Stage V
Pre-op.	0	9	10	14	0
At discharge	9	10	0	14	0

Stage IV patients remained unchanged, although their subjective parameters improved significantly (Chi-square analysis; *p* < 0.001).

All stage III patients became either Stage II or Stage I with a significant improvement in their pain free maximum interincisal opening values and subjective parameters (Paired t test; *p* < 0.001 and Chi-square analysis; *p* < 0.001 respectively).

Five stage II patients remained unchanged while 4 patients became stage I or free of symptoms. Patients that were Stage I at the beginning were not included in the study and there were no stage V patients within the study population.

Pre-operative Wilkes staging according to MRI data showed that 9 patients were Stage II with early signs of disc deformity in a slightly forward position. Ten patients were Stage III with a reducing anterior disc displacement and thickening of their discs. Fourteen patients were Stage IV with a non-reducing anterior disc displacement, moderate deformity and thickening of their discs. The bony changes were detected in Stage III and IV patients ranging from beaking of the condyle to abnormal bone contours around the articular eminence. Stage I and Stage V joints were not detected.

Arthroscopic examination revealed that 1 Wilkes Stage II patient had anterior disc displacement; 3 Wilkes Stage III patients had mild and localised thin fibrillations of the articular cartilage with antero-medial disc displacement; 5 Wilkes Stage IV patients had extensive, thick fibrillations of the articular cartilage with antero-medial disc displacement and creeping synovitis.

Findings of pre-operative clinical examination, pre-operative MRI and arthroscopic lysis and lavage were parallel; thus clinical, MRI and arthroscopic (under direct vision) Wilkes Staging of the 9 patients were identical.

When AAOMS success criteria [15] are considered, surgical treatment outcome in our group of patients is given in Table 6. The list of AAOMS criteria and the results applied to our study are below,

A. Masticatory function was improved in all, but 2 patients
B. Level of pain was of little or no concern for all, but 2 patients as measured by the modified visual analogue scale.
C. Mastication, deglutition, speech and oral hygiene hence mandibular function improved in all, but 7 of our patients had extended disability.
D. All of the patients had stable occlusion with no temporary or permanent premature contacts.
E. Recovery was uneventful and introduction of mouth stretching exercises immediately post-operatively limited period of disability.
F. No permanently disabling complications were encountered (further morbidity was limited).
G. Understanding and acceptance by patient (family) of favourable outcomes, known risks and complications is an imperative component of the informed consent process for surgical management of TMJ patients.
H. Symptoms and quality of life improved for most of the patients’ through out the follow up period.

**Table 6:** Treatment outcome according to the AAOMS (American Association of Oral and Maxillofacial Surgeons) criteria (2017)

Treatment outcome	Number of patients (n)
Excellent	14
Good	17
Poor	2
Success rate	94%

## Discussion

The contemporary evidence based management of ID with or without OA include; no treatment, medical treatment, physical therapy, arthrocentesis and arthroscopic surgery [15–20]. Treatment costs are quite variable from none (no treatment) to several thousand British pounds (arthroscopic surgery under general anaesthesia). One might think that not treating patients would not involve any costs and all the patients would eventually become asymptomatic as shown by Schiffmann et al. [16]. “No treatment” can actually be more costly, because of the extended distress of the patients, resulting in loss of labour and prolonged use of analgesics. For this reason the authors of this manuscript believe that not offering treatment to symptomatic ID with or without OA cases is unethical because of the psychological burden this would place on the patients. However “no treatment” should definitely be an option to be discussed with the patient during the informed consent process.

Information gathered from clinical and radiological examinations might fail to point towards a best treatment approach; hence deciding on the most appropriate treatment option for ID is difficult. The reliability of panoramic radiography is known to be poor for detecting OA or other soft tissue pathologies of TMJ. However MRI is a reliable tool inorder to detect bone marrow oedema, disc displacement with or without reduction, increased fluid level in the joint and computerised tomography (CT) is reliable for radiological diagnosis of OA [21].

CT obviously yields valuable data regarding the osseous structures and Ahmad et al. [21] suggests that clinical or research studies of OA should use CT when possible, however we are focused on the symptoms of the patients and a CT scan would not have changed our treatment modality and therefore was not deemed to be necessary before arthrocentesis and/or arthroscopy routinely. Panaromic radiographs taken in the present study served as a screening tool for TMJ pathology. Presence of osteophytic lipping, subarticular radiolucent areas, and presence of remodelling of the condyle in panoramic films are findings leading to the diagnosis of OA, however because of the low sensitivity of panaromic films in detecting OA, one should consider CT or cone-beam computerised tomography (CBCT) for optimal diagnostic evaluation of bony changes of TMJ [21].

Treating patients arthroscopically while arthrocentesis or conservative treatment would be sufficient will result in a rise of treatment costs and exposure of the patients to unnecessary risks. Therefore we preferred a stepped approach to manage symptomatic ID patients that is also suggested by Schiffman et al. [17]. We acknowledge that assessment of patients with pain from both joints can be confusing and therefore we included only unilateral cases. The fact that this is a retrospective study and therefore a comparison group is missing is a limitation of this study. A prospective, randomised controlled clinical trial aiming to compare the outcomes of various conservative and surgical treatment modalities in patients with internal derangement would be invaluable.

The subjective parameters were rated on a scale of 4 by the patient in the presence of the attending surgeon. We avoided using the VAS scale owing to the literacy status of some of our patients. The 4-point scale that was printed on paper was handed and read to the patient. Later the patient was asked to choose the appropriate number that best quantifies the subjective parameter in question. Mistakes in scoring were thus eliminated.

It is well known that appropriate selection of patients requiring surgical intervention is essential for successful treatment results [7, 22]. Moses [22] reported that 10 out of 400 patients with TMJ pain and dysfunction need surgical treatment (2.5%). In our study 1414 patients with TMJ pain and dysfunction attended oral surgery clinic during the study period, and 1371 of these were managed conservatively according to our standard treatment protocol. The remaining 43 patients received arthrocentesis and/or arthroscopy. Therefore 3% of our patients required surgical treatment, which is in accordance with the literature.

The follow-up period for 33 patients showed a normal distribution with a mean of 15.5 months. The details of the case notes revealed that most of the patients failed to attend their review appointments once they were satisfied by the result. All of the patients had access to the secretaries’ phones for booking a slot in the clinic if they thought it was necessary. Hence it could be argued that the duration of our follow-up period is adequate for a realistic and correct assessment of treatment outcomes. However we recognise that this is a limitation of the present study and studies with a prospective design can allow for longer follow-up periods.

The average increase in pain free inter incisal opening was 6 mm giving an average opening of 33 mm at discharge. Although this is lower then the normal values of inter incisal opening that is 35-45 mm and lower then the values achieved by Sorel et al. [23], the increase in our group is statistically significant. However clinical significance of the average increase in pain free inter incisal opening is debatable.

Ahmed et al. prospectively assessed the therapeutic benefits of arthroscopy and arthrocentesis in patients with internal derangement of their temporomandibular joints. They found that both treatment modalities improve mouth opening and reduce pain when conservative approaches have failed [24]. Emes et al. advocate that more invasive procedures should be considered for patients with TMJ pain who do not benefit from arthrocentesis [17]. Our findings are in accordance with these studies.

In their randomized controlled trial of 80 patients with arthralgia of the TMJ, Vos et al. showed that, arthrocentesis as initial treatment reduces pain and functional impairment more rapidly compared to conservative treatment. However, after 26 weeks, both treatment modalities achieved comparable outcomes [18].

Several studies report that arthroscopic TMJ lysis and lavage with or without anterolateral capsular release reduce arthralgia significantly. In accordance with these studies, our stepped treatment approach allowed a statistically significant improvement in our patients’ suffering as evaluated by “the subjective parameters” [25–29].

Staging of internal derangement of TMJ does not seem to be related to the severity of subjective parameters and the amount of relief of these symptoms after arthrocentesis and/or arthroscopy in our setting. Regardless of their staging, all patients benefit from arthrocentesis and/or arthroscopy particularly in pain relief. However pain free inter incisal opening of Stage IV patients appear to improve less than Stage II and Stage III patients. Thus it can be possible to speculate that, Stage II and III patients are more likely to have an improved mouth opening than Stage IV patients after treatment. The null hypothesis tested here can not be rejected within the limitations of this study, as subjective parameters and mouth opening of the patients improved at discharge. However randomized, prospective and controlled clinical studies should be performed for more evidence.

AAOMS success criteria [22] (“parameters of care” 1995) offered limited amount of data for comparison across studies. Therefore, in this study a revised version of AAOMS “parameters of care” that listed the “General Favorable Therapeutic Outcomes For Temporomandibular Joint Surgery” including arthrocentesis and arthroscopy was preferred [15] (Table 6).

Ideally VAS rating and where possible quantitative measuring of the above mentioned “General Favorable Therapeutic Outcomes” would enable comparison across groups and/or different studies. Similarly the “subjective parameters” rated on a scale of 4 in our study, allow temporal monitorization of the outcomes listed in the AAOMS publication [15].

Ahmed et al. reported that 2 patients out of 244 TMJ patients that were treated with arthroscopy or arthrocentesis had temporary weakness of the temporal branch of the facial nerve [24]. In our group no permanently disabling complications were encountered. However one of our patients experienced an inferior alveolar nerve block during arthrocentesis that completely resolved in an hour. This inadvertent local anaesthesia was thought to be due to a needle puncture on the medial wall of the joint capsule, and the local anaesthetic administered in the joint, must have infiltrated to the infra-temporal fossa. It is well known that mandibular nerve lies close to the joint and it is possible that the local anaesthesia was achieved through this mechanism. We are not aware of any similar reported complications during arthrocentesis. On the other hand brutal manipulation of the thoracars during TMJ arthroscopy may lead to temporary weakness of the Vth and VIIth cranial nerves [30]. Accordingly one of our arthroscopy patients had temporary weakness of her eyelid with complete recovery at the end of 3 weeks. She was treated conservatively with eye lubricants during this period. Other reported complications of this procedure that were not seen in our group include perforation of the external auditory meatus and extradural haematoma [24].

## Conclusion

The stepped approach for the management of symptomatic internal derangement with or without osteoarthritis is a predictable treatment strategy to reduce the suffering of our patients, hence improve the quality of their lives. However, it is imperative for the patients to understand the involved procedures and their outcomes, for developing realistic expectations and improving patient co-operation.
